# Early detection of colonic anastomotic leak

**DOI:** 10.1111/ans.19243

**Published:** 2024-10-01

**Authors:** Claudia Paterson, Andrew G. Hill

**Affiliations:** ^1^ Department of Surgery, Te Whatu Ora – Counties Manukau University of Auckland Auckland New Zealand

Anastomotic leak (AL) is a feared complication of colorectal surgery. This perspective is written to provide some thoughts on current research, with a focus on early detection of colonic AL prior to the leak becoming clinically relevant.

## The context

Technical failure or inadequate blood flow are obvious contributors to AL. However, many other factors can limit a patient's progress towards anastomotic healing, such as malnutrition, smoking, immunosuppression, and variations in perioperative care.^1^ AL results in some intraperitoneal contamination by enteric bacteria even if mechanical bowel preparation and antibiotics have been administered. A well‐studied enteric bacterium postulated to be involved in the pathophysiology of AL is *Enterococcus faecalis*. Some strains of *E. faecalis* can degrade collagen and activate tissue matrix metalloprotease‐9 (MMP9) in intestinal tissues.^2^ How the body reacts to contamination of enteric bacteria and stops progression to infection, how well the body can repair this leakage, and the inflammatory response appear to influence the clinical significance of AL and help in its early detection. Early detection allows for early rescue of the patient, decreasing the risk of significant morbidity and mortality from AL.

## Early detection of anastomotic leak

Following surgery, the environment around the anastomosis is awash with pro‐ and anti‐inflammatory cytokines as part of the inflammatory and healing process. In the uncomplicated situation the composition of this peritoneal fluid follows a predictable course over several weeks.^3^ Peritoneal fluid can be measured using a drain if in situ, although this is less common in the ERAS era. Inflammatory cytokines are reflected within the systemic circulation, albeit at lesser concentrations, and can be measured using blood tests.^3^ In AL this inflammatory environment is altered, and this appears to occur well before clinical features become apparent, perhaps as early as postoperative Day 1.^4^ Normal levels of inflammatory systemic biomarkers in the early postoperative period suggest that the anastomosis is intact and will remain so. It is possible to be confident in predicting that AL will not happen as soon as postoperative Day 3, but early detection is more challenging.^5^

Early postoperative elevations of CRP and IL‐6 have been shown to be useful for early detection of AL after elective colonic resection, and this seems to be most accurate between postoperative Days 3 to 5.^4^ Systemic levels of CRP, IL‐10, and IL‐6 are elevated in patients prior to AL being clinically detected, although their specificity is problematic.^5^, ^6^ Procalcitonin has also been investigated for its utility in early detection of AL. It is a biomarker predominantly used in the intensive care unit for distinguishing between viral and bacterial infection.^7^ The highest diagnostic accuracy for procalcitonin to detect AL is postoperative day 5.^7^ Other biomarkers have been investigated, and can be divided into three main categories: ischaemic, inflammatory, and microbiological. A systematic review from our research group identified 49 putative biomarkers to detect AL following colorectal resection and found that identified biomarkers had good negative predictive values but were poor positive predictors of AL.^3^ A non‐exhaustive list includes chemokines such as CXCL5 and CCL8, growth factors such as TNF‐a, matrix metalloproteinases such as MMP‐9, neutrophil‐to‐lymphocyte ratio (NLR), faecal calprotectin, and peritoneal lysosome function.^3^, ^8^, ^9^, ^10^, ^11^ Limitations of lesser‐known biomarkers include a prolonged analytic time and high costs.

## Other factors influence inflammation postoperatively

Clinicians need to be aware that several factors can alter biomarker profiles. Patients undergoing laparoscopy, compared to equivalent open surgery, have significantly reduced systemic IL‐6, neutrophils, and CRP early in the postoperative period.^12^ Patients with a BMI greater than 30 kg/m^2^ have significantly higher levels of CRP regardless of operative approach, and statins alter both preoperative and postoperative inflammatory markers.^12^

Thus, the postoperative inflammatory response is influenced by surgical approach, perioperative medications, and patient factors. These findings have important implications in the utility of biomarkers in the diagnosis of postoperative surgical complications, particularly in the early diagnosis of AL.

## Rectum versus colon

It is common for studies in AL to include both rectal and colonic surgery and make no distinction between the two. This is problematic and makes the interpretation of data difficult. Factors that need to be considered are different anatomy, different rates of leak, different consequences of leaks, rates of defunctioning stomata, and different inflammatory cytokine profiles.^13^ The Rahbari grading system is based on rectal surgery and is probably not applicable to colonic surgery.^14^

## Combinations of clinical parameters and biomarkers

Multiple clinical parameters are associated with AL following colonic surgery. These vary in their time of onset and their strength of association. Elevations of heart rate, respiratory rate, and temperature are commonly associated with AL and may aid in its early detection, with heart rate and respiratory rate showing particularly good positive predictive values for AL.^15^

It should be possible to combine clinical parameters, cytokines, CRP, procalcitonin, and white cell count, taking into account factors such as obesity and site of surgery to provide a more accurate diagnosis of AL prior to it becoming clinically apparent.^3^ It is likely that artificial intelligence will be useful in future studies, via machine learning algorithms. For instance, a web‐based risk calculator has been developed to predict which patients are at high risk of AL.^16^

## Selective use of CT according to risk categorization

The aim of any scoring system developed from these parameters should be that patients can be put into one of three groups in the first few days after surgery. The first group are those in whom clinical suspicion is low. These patients should be observed and discharged as soon as they have met discharge criteria. The second group is those who clearly have AL and require prompt intervention. The third group is those in whom there is equipoise. In this case an appropriate investigation, usually a CT with luminal contrast, will guide management.^17^

In conclusion, the time is near where early diagnosis of AL following colonic surgery should be routine. This should allow minimally invasive interventions to be implemented prior to the patient requiring emergency intervention, a stoma that may end up being permanent, and a requirement for prolonged intensive care admission.

## Author contributions

**Claudia Paterson:** Investigation; methodology; writing – original draft; writing – review and editing. **Andrew G. Hill:** Conceptualization; investigation; supervision; writing – original draft; writing – review and editing.

## Funding information

Claudia Paterson is funded by the Health Research Council on a Clinical Research Training Fellowship. Andrew Hill is an Editorial Board member of the ANZ Journal and a co‐author of this article. To minimize bias, he was excluded from all editorial decision‐making related to the acceptance of this article for publication.
